# Swarming Motility Without Flagellar Motor Switching by Reversal of Swimming Direction in *E. coli*

**DOI:** 10.3389/fmicb.2020.01042

**Published:** 2020-05-21

**Authors:** Zhengyu Wu, Rui He, Rongjing Zhang, Junhua Yuan

**Affiliations:** Hefei National Laboratory for Physical Sciences at the Microscale and Department of Physics, University of Science and Technology of China, Hefei, China

**Keywords:** swarming, bacterial flagellar motor, bacterial motility, surface effect, run and tumble

## Abstract

In a crowded environment such as a bacterial swarm, cells frequently got jammed and came to a stop, but were able to escape the traps by backing up in their moving course with a head-to-tail change (a reversal). Reversals are essential for the expansion of a bacterial swarm. Reversal for a wildtype cell usually involved polymorphic transformation of the flagellar filaments induced by directional switching of the flagellar motors. Here we discovered a new way of reversal in cells without motor switching and characterized its mechanisms. We further found that this type of reversal was not limited to swarmer cells, but also occurred for cells grown in a bulk solution. Therefore, reversal was a general way of escaping when cells got jammed in their natural complex habitats. The new way of reversal we discovered here offered a general strategy for cells to escape traps and explore their environment.

## Introduction

Bacteria with peritrichous flagella, such as *Escherichia coli*, swim in aqueous environment by rotating flagella, each driven by a direction-switchable rotatory motor (flagellar motor) at its base (Berg, 2003). Switching of motor rotation is regulated by the signal protein CheY after phosphorylated by the kinase CheA in the bacteria chemotaxis system (Stock et al., 1989; Welch et al., 1993; Sourjik and Berg, 2002). The direction of motor rotation determines the bacterial swimming mode. When all motors rotate counterclockwise (CCW), their flagella form a bundle, and the cell swims smoothly (called “run”) (Berg and Anderson, 1973; Silverman and Simon, 1974; Macnab, 1977). When one or more motors switch to clockwise (CW) rotation, the associated flagella escape from the bundle while the cell turns to a new direction, producing a “tumble” (Turner et al., 2000). As a motility organelle, flagellum is formed by 11 protofilaments (Asakura, 1970; Samatey et al., 2001; Shibata et al., 2005), existing in different polymorphic forms (Calladine, 1976, 1978; Kamiya et al., 1979; Turner et al., 2000). Each flagellum is several micrometers long and rotates at a rate ∼100 Hz (Berg, 2008). The polymorphic forms can switch from one to another, when motor switches between CCW and CW (Turner et al., 2000).

Swarming is a motility form of flagellum-driven bacteria moving across solid surfaces in a group (Kaiser, 2007; Copeland and Weibel, 2009; Darnton et al., 2010; Kearns, 2010; Patridge and Harshey, 2013; Belas, 2014). *E. coli* can swarm on soft Eiken agar surface with proper moisture and rich nutrients, like many other peritrichous bacteria (Harshey, 1994; Harshey and Matsuyama, 1994). Motile behavior of bacteria when swarming on wet surface is different from that when swimming in bulk liquid. When swimming in bulk aqueous media, *E. coli* explores the environment in a random walk pattern of alternating run and tumble, and the turn angle of tumble of *E. coli* swimming in free space is a skewed distribution with an average value of about 60° (Berg and Brown, 1972; Turner et al., 2000). When swarming on a wet surface, bacteria rarely perform a tumble (Darnton et al., 2010), they instead are continually reoriented by colliding with neighbors, and back up in their course with a head-to-tail interchange (a reversal) when their motion is stopped by neighbors. Reversals are essential for bacteria to swarm on a surface. It is believed that reversals help cells to escape from confined environments (Cisneros et al., 2006), complete cell alignment, and increase the outflow of the cells across the edge of the swarm (Wu et al., 2009), thereby making swarm possible. A previous study found that reversals of *E. coli* in swarm were accompanied with filament polymorphic form transforming from the normal to curly state, triggered by motors switched from CCW to CW rotation (Turner et al., 2010). For a Δ*cheY* strain, the filaments will not undergo polymorphic transformation as the flagellar motor does not switch direction. Nevertheless, it was found that smooth swimmers (Δ*cheY*) of *Salmonella typhimurium* partially restored swarming motility when enough water was sprayed to the swarm plate to achieve high moisture (Wang et al., 2005). Therefore, it is intriguing how smooth-swimming bacteria escape from obstacle or traps and whether these bacteria reverse in a swarm. Here, we found that a smooth swimmer with Δ*cheY* can swarm on Eiken agar surface when the surfactant Tween 20 was added with appropriate concentration, and that the smooth swimmers also showed high frequency of reversals. By fluorescence labeling of the filaments and by observing their behavior during reversals, we discovered a new reversal mechanism—reversal without motor switching.

## Results

### Δ*cheY* Mutant Swarms on Soft Agar Supplemented With Surfactant

Under general swarm assay conditions for wildtype *E. coli*, any strain with defective *che* gene cannot swarm on the surface (Harshey and Matsuyama, 1994), unless sufficient wetness was provided to the surface. To increase surface wetness, one way was to spray water on the surface directly, which was proved to be a way to partially restore swarming motility of *S. typhimurium* and *E. coli* with Δ*cheY* (Wang et al., 2005; Ford et al., 2018). Another way was to add surfactant, such as surfactin or Tween 80. *E. coli* wildtype cells achieved swarming on the Difco agar (less moisture than the Eiken agar) plate with these two surfactants, but not without them (Toguchi et al., 2000; Niu et al., 2005). Here we used Tween 20 (a non-ionic surfactant like Tween 80) as the surfactant and incorporated it into Eiken swarm medium to further enhance the wetness of the surface. To determine the optimal concentration for Tween 20, we inoculated HCB1736 (Δ*cheY*) on swarm plates with 0.01, 0.05, 0.1, 0.5, or 1% Tween 20. As shown in Supplementary Figure S1, the optimal concentration was 0.05%; hence, we used this condition to implement the following experiments. To rule out the possibility of motility-independent expansion (e.g., driven by the outward pressure due to growth) on swarm plates with Tween 20, swarming by motile (wildtype or Δ*cheY*) and non-motile (Δ*motB*) strains were compared on swarm plates with or without 0.05% Tween 20. There was no swarming expansion of the non-motile strain on either plate (Supplementary Figure S2).

We observed the swarm plate of the Δ*cheY* strain under microscope. In the region near the edge of a swarm, the swarm plate could be divided into three areas similar to that of the wildtype *E. coli* (Wu and Berg, 2012), as shown in Supplementary Figure S3A. Monolayer of cells moved collectively at the leading edge, and after a transition region, it gradually turned into multiple layers, as illustrated in Supplementary Figures S3B–D. There were also some cells stuck on the surface sparsely ahead of the leading edge. We measured about 50 cells in each motility area. Cells in monolayer area were 4.53 ± 1.01 μm (mean ± std) long on average, about twice the length of cells in multilayer area and cells grown in a dilute aqueous medium (e.g., TB solution with 1% Bacto tryptone and 0.5% NaCl), which were 2.55 ± 0.81 and 2.75 ± 0.72 μm, respectively. The distributions of cell length are shown in Supplementary Figure S4. The average number of filaments on a Δ*cheY* swarmer cell (in monolayer) was 4.70 ± 1.97, with average length of 5.24 ± 2.07 μm. The Δ*cheY* swarmer cells had slightly more filaments, but with similar filament length, compared to cells grown in TB solution (3.68 ± 1.28 filaments per cell and average length of 5.19 ± 1.66 μm, respectively). The distributions of filament number and length are shown in Supplementary Figure S5. The Δ*cheY* swarmer cells had fewer filaments, but with similar filament length, compared to wildtype *E. coli* swarmer cells (7.6 ± 3.0 filaments per cell, with average length of 4.5 ± 2.0 μm) as described previously (Turner et al., 2010).

We compared the swarming of the Δ*cheY* mutant strain HCB1736 and the wildtype strain HCB1. The sizes of the swarm rings were very different between the wildtype and Δ*cheY* mutant strains after the same amount of incubation time, with the ring of the wildtype always larger than that of the mutant under same conditions (described in section “Materials and Methods”). We measured the advancing rates of the swarm front of the two strains, each measured for 10 swarm plates, and obtained average rate of 0.82 ± 0.13 and 4.75 ± 0.38 μm/s (mean ± std) for the Δ*cheY* strain HCB1736 and the wildtype strain HCB1, respectively. To check the difference in the motility of individual bacteria on the swarm plates for each strain, tracking assay was performed on the swarm plates. We fluorescently labeled some of the cells in the swarm by constitutively expressing mCherry, so that the density of the fluorescent cells was sparse enough for us to track individual cells. There was no significant difference in the cell velocities in the monolayer region (27.17 ± 6.73 μm/s from 377 traces of the Δ*cheY* strain, and 30.87 ± 7.32 μm/s from 517 traces of the wildtype strain). The mean velocity of the Δ*cheY* strain in multilayer area was 29.49 ± 8.57 μm/s, similar to that in the monolayer area. The distributions of cell velocities are shown in Supplementary Figure S6. In addition, we calculated the cell propulsion angles (the angle between the major axis of a cell body and its velocity vector) of the three cases (Δ*cheY* strain monolayer area, wildtype strain monolayer area, and Δ*cheY* strain multilayer area), resulting in similar values for the three cases (28.34 ± 5.12°, 29.48 ± 4.86°, and 32.72 ± 5.36°, respectively). The distributions of propulsion angles are shown in Supplementary Figure S7. To test whether there was a difference in growth rate between the two strains, we measured the growth curves for the two strains in TB solution at 30°C, and found that they were similar (Supplementary Figure S8). We analyzed the trajectories for the wildtype and Δ*cheY* strains, and found that both exhibited normal diffusive behavior, with linear relationship between the mean squared displacement (MSD) and the time lag (Supplementary Figure S9). The diffusion constant for the wildtype (29.4 μm^2^/s) is larger than that for the Δ*cheY* strain (17.1 μm^2^/s). The distributions of waiting times between turning points on the trajectories are exponential shape (Supplementary Figure S10), consistent with the normal diffusive behavior. This suggested that the swarming behavior of *E. coli* may be different from that of *Bacillus subtilis* and *Serratia marcescens* which exhibited Levy walk behavior (Ariel et al., 2015).

The swarms of the wildtype and the Δ*cheY* strains normally had a similar structure that included monolayer, transition, and multilayer regions. By comparing the cell density of each region, we found that cells in the transition/multilayer regions were too dense to allow clear visualization of the dynamic of flagella, so we paid more attention to the behavior of the cells in the monolayer region in subsequent experiments.

### Reversals in Swarms Without Motor Switching

**FIGURE 1:**
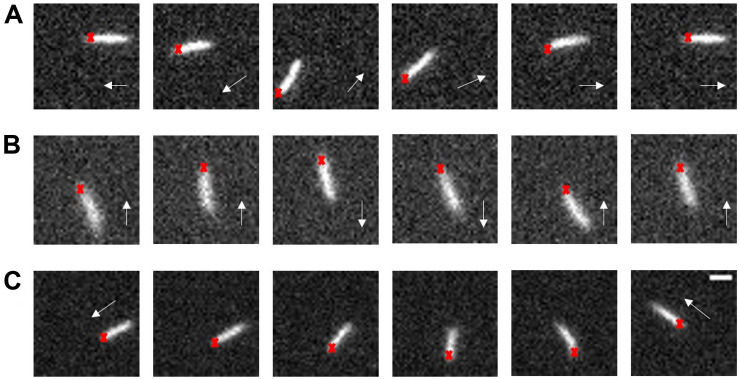
Typical swarm cell reversal on the swarm agar plate of the Δ*cheY* strain. **(A)** The cell reversed without changing the orientation of its long axis, Δangle ∼π. **(B)** The cell exhibited successive reversals, Δangle ∼ 0. **(C)** The cell body underwent a significant rotation, Δangle depended on the degree of the body rotation. The red crosses represent the cell head at the beginning and the white arrows indicate the instantaneous velocity direction of the cell, scale bar = 3 μm.

When tracking the fluorescence labeled cells in swarms, we found that Δ*cheY* cells surprisingly also performed reversals. Reversal phenomena were confirmed by examining the tracking videos frame by frame. We calculated the reversal frequency and found a reversal frequency of 0.375 s^–1^ for the Δ*cheY* strain in the monolayer region (total cells = 352, total length = 954.72 s) and 0.4 s^–1^ for the wildtype strain (total cells = 392, total length = 1104.92 s). So there was no significant difference in reversal frequency between the two strains. This analysis was performed on all tracks that lasted longer than 2 s, regardless of whether reversals occurred. Nearly 66% of the tracks for the Δ*cheY* strain (number of cells = 232) had at least one reversal events during the average tracking time of about 3 s per track. Such a large proportion indicates that reversal is a common motility mode in bacterial swarm. We recorded about 100 videos of reversal events on swarm plates and measured the reversal angles. Similar to the wildtype, the angular change of most of the Δ*cheY* strain reversal events were about 100∼180°, during which the cell backed up and the orientation of its body axis remained almost unchanged. For the other reversal events with small angular changes, the cell body underwent a significant spin or the cell exhibited successive reversals like the wildtype (Turner et al., 2010). Some of the cases are shown in Figure 1 and Supplementary Movies S1–S3. The distribution of reversal angles is shown in Supplementary Figure S11.

Previous studies on flagella dynamics of swarming bacteria found that *E. coli* cells backed up with flagella filaments transforming from normal to curly state, which required motor switching (Turner et al., 2010). To explore how the Δ*cheY* cells (HCB1736) performed reversals without motor switching, we sought to visualize the dynamics of flagella filaments for the Δ*cheY* cells in swarms. We stained the filaments with fluorescent dyes and imaged them in a sandwich-like setting described previously (Turner et al., 2010) (see details in section “Materials and Methods”).

To investigate the flagella dynamics during Δ*cheY* cell reversals, we examined the reversal events recorded in our videos frame by frame. Most of the cases can be generalized as below. As the cell got jammed and came to a stop, the filament bundle became less stable, and rolled CW from the cell tail (defining head/tail using the original moving direction of the cell before stopping) due to hydrodynamic surface effect of the agar surface (Wu et al., 2011). As the filaments rolled to the side of cell body, they pushed the cell body to rotate about the pinned cell head if there was spatial freedom to rotate, otherwise the filaments kept rolling CW to the cell head without much of cell body rotation. All of the reversal processes were accomplished without flagella polymorphic transformations. Two of the representative cases are illustrated below.

**FIGURE 2:**
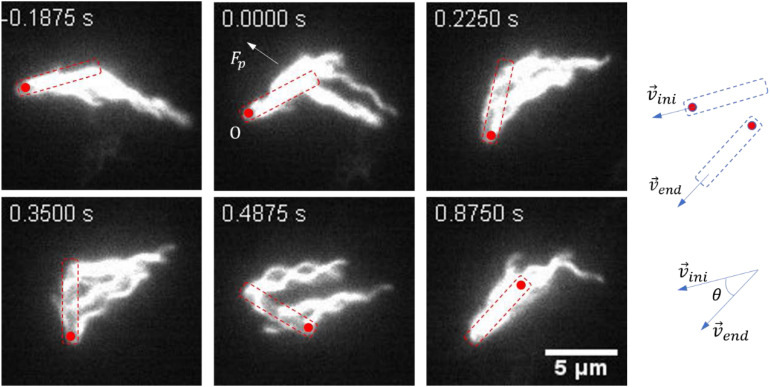
Images sequences taken from Supplementary Movie S4. The time stamp of each movie frame was shown. In this case, multiple flagella filaments, when rolled to the side of the cell, provided lateral force (*F*_p_). With the pinned head as the instantaneous fixed origin (O), *F*_p_ leads to a torque on the cell body that causes it to rotate CCW about O, in order for the cell to find the suitable direction to escape (usually with a small angle change of the moving direction). The cell body is indicated by a dashed box, and the red circle marks the position of the initial head. The small change in angle is schematically shown on the right.

A representative reversal case with large angular rotation of the cell body is shown in Figure 2 and Supplementary Movie S4. At *t* = − 0.1875 s, the bacteria moved from right to left with the filaments bundled at one end of the cell body (defined as cell tail). At *t* = 0 s, the cell got jammed and came to a stop, and the filaments began to roll CW. From 0 to 0.4875 s, filaments rolled to the side of the cell body and pushed it to rotate about the pinned cell head, and the filaments gradually gathered at the cell head as the filaments rolling CW and the cell body turning CCW. At *t* = 0.875 s, the filaments finished re-bundling at the cell head as the cell found a suitable direction to move, and a reversal was completed. This usually resulted in a small angle change in the moving direction.

**FIGURE 3:**
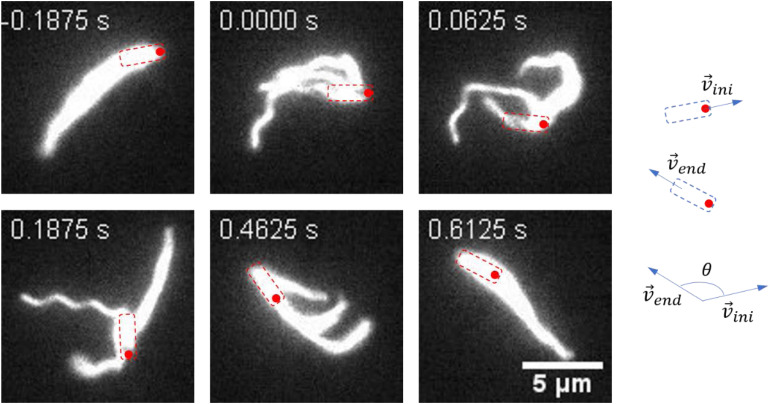
Images sequences taken from Supplementary Movie S5. The time stamp of each movie frame was shown. In this case, the filaments rolled from one end of the cell body to the other end and re-bundled. This process was usually accompanied with a large angle change of the moving direction. The cell body is indicated by a dashed box, and the red circle marks the position of the initial head. The large change in angle is schematically shown on the right.

A representative reversal case with small angular rotation of the cell body is shown in Figure 3 and Supplementary Movie S5. At *t* = − 0.1875 s, the bacterium moved from the left to the right with filaments bundling at the tail. At *t* = 0 s, the cell got jammed and the filaments started to unbundle and roll CW. The next two pictures (*t* = 0.0625 s and *t* = 0.1875 s) showed that the filaments gradually rolled to the cell head while the cell position remained almost unchanged. At *t* = 0.4625 s, all the filaments rolled to the cell head and ready to bundle. At *t* = 0.6125 s, the cell found a suitable direction to move, the filaments re-bundled at cell head, and the bacterium moved to the left. This usually resulted in a large angle change in the moving direction.

As expected, the filament polymorphic form was always in the normal state during reversal events observed here, because there was no motor switching in Δ*cheY* mutant. Reversal events reported previously in wildtype *E. coli* swarming always involved polymorphic transformation of the filaments (Turner et al., 2010), and reversal events without motor switching were not identified, probably because for the wildtype cells reversal with filament polymorphic transformation was an easier process with lower energy barrier than reversal without filament polymorphic transformation. Nevertheless, the fact that the Δ*cheY* and wildtype strains in the monolayer area on a swarm plate showed similar reversal frequencies, suggested that the reversal frequency was determined by how frequently the cells were stopped by neighbors. This led to a further prediction: as cell density increases, leading to higher tendency for the motion of the cells to get blocked by neighbors, the cell reversal frequency would increase.

### Reversal Frequency Increased With Cells Density

To test the prediction, we explored the relationship between cell density and reversal frequency in a 2d environment. It was shown that the swarm–air interface of an *E. coli* swarm was stationary (Zhang et al., 2010), and placing a polydimethylsiloxane (PDMS) sheet above the agar plate did not affect the spreading rates of swarm edges (Turner et al., 2010). To vary the cell density, we added a 2.5 μL drop of swarm medium to the swarm edge, and placed a PDMS sheet over the swarm edge. We found that the density of bacteria near the medium dropping point was the lowest, and the density gradually returned to the original level as the distance from the dropping point increased. Therefore, we could track the trajectories of individual cells with fluorescent imaging at different densities and then analyzed the reversal frequency at each cell density.

**FIGURE 4:**
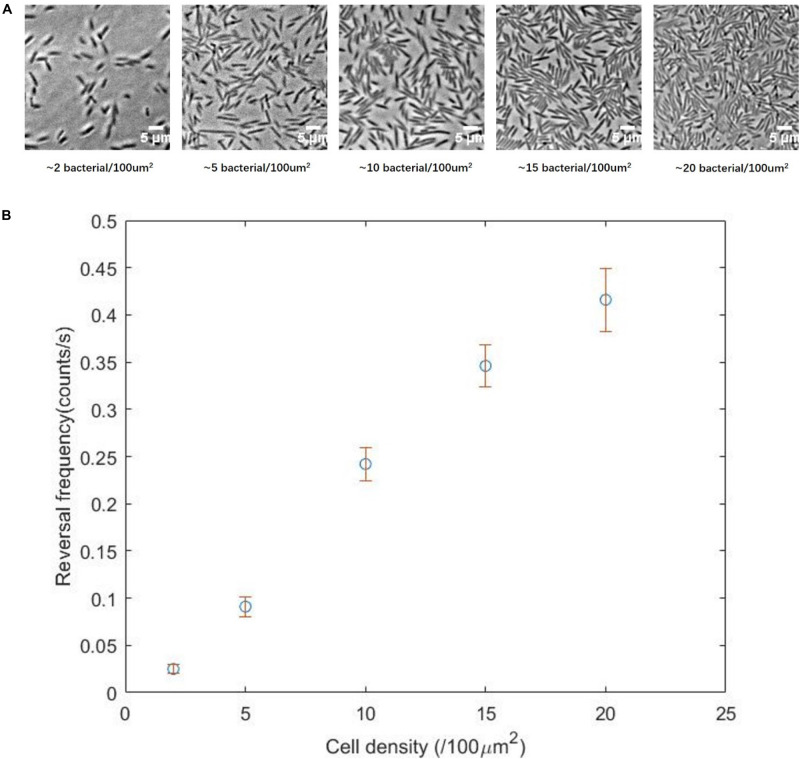
**(A)** Two-dimensional movement of the Δ*cheY* strain at different cell density, showing the transition from single-cell state to normal swarming state. **(B)** Reversal frequency as a function of the cell density, showing a clear correlation between the two parameters.

We divided the cells densities into five levels: 2, 5, 10, 15, or 20 cells per 100 μm^2^ (Figure 4A). These five densities from low to high characterized the transition from a single-cell state to the normal state of the swarm phase. Subsequently, the reversal frequencies at different density levels were measured. We found that the higher the cell density, the higher the reversal frequency (Figure 4B), consistent with our prediction. The cell density in the monolayer region on a normal swarm plate is usually more than 15 cells per 100 μm^2^, corresponding to the fourth or fifth panel of Figure 4A.

### Reversal Without Motor Switching for Cells Grown in Bulk Liquid

**FIGURE 5:**
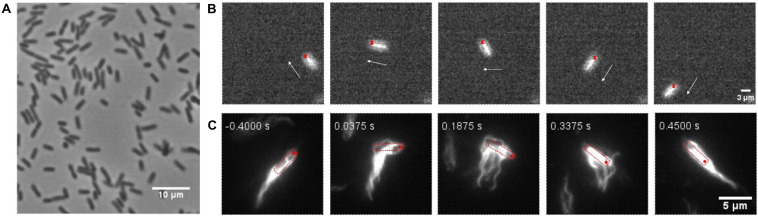
**(A)** Bright-field image of cells in a quasi-two-dimensional water layer. **(B)** A typical cell reversal in a quasi-two-dimensional environment for Δ*cheY* cells grown in bulk TB solution. Image sequences were taken from Supplementary Movie S6. The red cross represents the head at the beginning and the white arrow indicates the instantaneous velocity direction of the cell. **(C)** Flagellar dynamics of a cell (grown in bulk TB solution) undergoing reversal. Image sequences were taken from Supplementary Movie S7. The cell body is indicated by a red dashed box, and the red circle marks the position of the initial head.

Cell reversals were usually found with swarmer cells which exhibited clear morphological differences (longer cell body, more filaments, etc.) from cells grown in bulk liquid. In naturally occurring complex environments such as soil or biological tissues, bacteria would get jammed from time to time, and cell-reversal should be a general way to escape. So we hypothesized that the reversal mechanism we found here should not be limited to swarmer cells. We tested whether cells of a Δ*cheY* mutant grown in bulk liquid (e.g., TB solution) could also reverse the moving direction. We used a coverslip and a PDMS sheet to construct a quasi-two-dimensional water layer with a suitable concentration of bacteria (Figure 5A, see details in section “Materials and Methods”), to present an environment with possible cell stopping by neighbors. By fluorescently labeling cell bodies and flagella staining, we could clearly observe cell swimming and the corresponding flagellar dynamic behavior.

We observed cell reversal in this environment (Figures 5B,C and Supplementary Movies S6, S7). Compared with a swarm plate, the density of bacteria in the field of view was greatly reduced (∼5 cells per 100 μm^2^, similar to the cell density in the second panel of Figure 4A), so cells had greater spatial freedom to reorient the cell body. From the observed reversal examples, the filaments usually rolled to the side of the cell body, and drove the cell body to rotate. This was consistent with one of the reversal modes observed in swarm. Since it was difficult to artificially construct a two-dimensional environment with high bacterial density in which some cell bodies would be completely jammed without freedom of reorientation, we had not observed the other reversal mode in which the cell body did not rotate. Nevertheless, it is clear that the reversal mechanism we discovered here is a general way of escaping for *E. coli*, which does not depend on whether they are swarmer cells or not.

## Discussion and Conclusion

By adding suitable concentration of the surfactant Tween 20 to swarm plate, we found that the Δ*cheY* mutant strain HCB1736 could swarm on the Eiken agar surface. The surfactant supports the swarming of Δ*cheY* cells probably by reducing the surface tension at the outer edge of the swarm that would have restricted the spread of a swarmer colony. We characterized the morphological characteristics of different regions of the swarm plate, and studied the motility properties of bacteria in the corresponding regions by tracking individual fluorescent bacteria. Moreover, we compared the motility of the wildtype and Δ*cheY* mutant strains on surfactant-containing swarming plates, and found that there was no significant difference in the motility parameters of individual bacteria (including swimming speed, propulsion angle, and reversal frequency). But the spreading rates of the swarm edge differ significantly, probably due to increased surface wetness on the swarm plate with the wildtype strain with motor switching (Mariconda et al., 2006).

**FIGURE 6:**
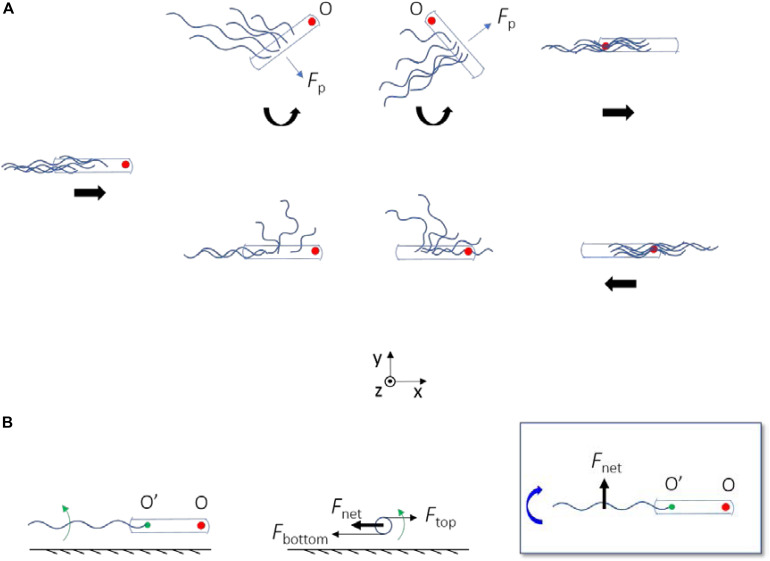
**(A)** A schematic diagram depicting the two ways by which Δ*cheY* mutant can reverse without motor switching. The straight thick arrows below the cells indicate the velocity direction of the bacterium, and the curved ones represent the direction of rotation of the cell body about the pinned head (O). When there is no arrow, the bacterium is at rest. The red filled circle marks the position of the initial head. In both cases, the flagella roll CW about their ends on the cell body. The flagella generate a propulsion force on the cell body (*F*_p_). When there is spatial freedom for the cell body to rotate, *F*_p_ leads to a *z*-torque on the cell body that makes it rotate CCW about O (top panels); otherwise the cell body remain fixed (bottom panels). **(B)** Physical mechanism for CW rolling of the flagella near surface. Left, center, and right panels are the side, rear, and top views, respectively. Green arrows represent the CCW rotation of the flagellum about its axis (viewed from rear). The green filled circle marks the end of the filament on the cell body (O’). *F*_net_ with O’ as the instantaneous fixed origin leads to a negative *z*-torque on the flagellum, which makes it roll CW about O’ (as denoted by the curved thick blue arrow).

By observing the trajectories of individual cells on the swarm plate, we confirmed that high frequency of reversals occurred. Reversals occurred on average every 2.5 s for each cell and required about 0.5 s for completion, resulting in a jammed state that occupies about 20% of the time period for each cell. This demonstrated that cells have a high probability of becoming jammed in swarm, and that reversal is a common motility mode in bacterial swarm even for Δ*cheY* mutant strains. Reversals might contribute to the chemotaxis behavior of bacteria at high cell density (Colin et al., 2019). Fluorescently labeling flagellar filaments made it possible to visualize the dynamic behavior of the filaments during the reversal process. Through analysis of the reversal events, we discovered new mechanisms for *E. coli* reversal without motor switching (Figure 6A). When a cell got jammed and came to a stop, its filament bundle became unstable and rolled CW about the ends of the filaments on the cell body (Figure 6B), then two possible cases occurred: when the filaments rolled to the side of the cell body, they generated a lateral force that cause the cell body to rotate CCW about its pinned head, and the filaments were re-bundled to complete the reversal when the rotation angle was appropriate (Figure 6A top). On the other hand, if there was no freedom of rotation for the cell body, the filaments kept rolling to the other side of the cell body, and re-bundled to complete the cell reversal (Figure 6A bottom). Reversal behavior produced by the combination of the above two cases was also observed.

A previous study showed that flagella of a wildtype *E. coli* swarmer cell stuck near a surface, can roll CW around the cell body if the motor rotation is CCW, thereby driving a CW fluid flow around the cell body (Wu et al., 2011). Similar mechanism for generating CW fluid flow around stuck cells of *B. subtilis* has been proposed (Cisneros et al., 2008). Here, we demonstrated an amazing maneuver that the cells can carry out using similar dynamics of the flagella: they can reverse the moving direction to escape from the traps by flagella rolling to the appropriate position with/without simultaneous cell body rotation.

Several species of bacteria use instability of the flagellar filament to escape from traps, by wrapping the filament around the cell body and so that the cells back out from the traps (Kuhn et al., 2017; Constantino et al., 2018; Kinosita et al., 2018). Similar to the case of wildtype *E. coli*, all of the cases involved switching of the flagellar motor. In the reversals observed in this study that did not involve motor switching, the flagellar instability is exemplified as the CCW-rotating flagella (viewed from the distal end) rolling CW near a surface (viewed from above) without motor switching. This is due to hydrodynamic effect of the surface, as illustrated in Figure 6B. As the flagella rotate CCW near the surface, bottom parts of the flagella are located close to the surface and top parts are located further away. The viscous drag coefficient decreases with increasing distance from the surface. Thus, the bottom parts are subjected to a larger viscous drag compared to the top parts, resulting in a net viscous drag that induces CW rolling of the flagella (Figure 6B). This is similar to one of the mechanisms that make *E. coli* swim in CW circular motion near a surface (Diluzio et al., 2005; Lauga et al., 2006).

Reversal for a wildtype *E. coli* cell (with motor switching) usually involved polymorphic transformation of the filaments (Turner et al., 2010). Here, we discovered a new way of reversal without filament polymorphic transformation for cells without motor switching. In nature, diverse bacteria live everywhere, and many of them need to navigate complex porous environment such as soils and sediments (Dechesne et al., 2010; Scharf et al., 2016), or tissues and biological gels (Harman et al., 2012; Ribert and Cossart, 2015; Datta et al., 2016). They frequently get trapped by constrictions when moving through these environments (Bhattacharjee and Datta, 2019), and naturally try to back out by reversals. As bacteria exhibit diverse ways of motility, many bacteria do not show motor switching-induced polymorphic transformation of flagella (Jarrell and Mcbride, 2008; Krell et al., 2010). For those bacteria, they nevertheless are still able to escape from the traps by reversals without motor switching. Therefore, the new way of reversal we discovered here, offered a general strategy for cells to escape traps in their complex natural habitats.

## Materials and Methods

### Strains and Plasmids

*Escherichia coli* strain AW405 (HCB1) is wildtype for chemotaxis. HCB1736 (Δ*cheY*) and ZW1 (Δ*motB*) are derivatives of AW405. The filament gene *fliC* was further deleted from HCB1736, yielding SM2. The plasmid pBAD33FliC^S353C^ expresses FliC^S353C^ under the control of an arabinose-inducible promoter in the vector pBAD33. The plasmid pTrc99amCherry expresses mCherry under control of an isopropyl-β-D-thiogalactoside (IPTG)-inducible promotor in the vector pTrc99a. HCB1 and HCB1736 transformed with the plasmid pTrc99amCherry were used for tracking of bacterial trajectories. SM2 carrying pBAD33FliC^S353C^ was used for visualizing flagella.

### Cell Culture

A single-colony isolate was grown in 3 mL of LB solution (1% Bacto tryptone, 0.5% yeast extract, and 0.5% NaCl) overnight to saturation on a rotary shaker (200 r/min) at 33°C. Dilutions of this culture were used to inoculate swarm plates or liquid cultures. For growth in bulk liquids, all strains (with a dilution of 100 × from the overnight culture) were grown in 10 mL of TB solution (1% Bacto tryptone, 0.5% NaCl) to an OD_600_ between 0.45 and 0.50. Appropriate antibiotics (25 μg/mL chloramphenicol for the pBAD33 vector and 100 μg/mL ampicillin for the pTrc99a vector) and the appropriate inducers (0.2 mM IPTG for mCherry expression and 0.01% arabinose for FliC expression) were added into the culture if needed.

### PDMS Sheet

Polydimethylsiloxane (Sylgard 184; Dow Corning) was prepared according to the manufacturer’s specification and spun onto a 10 cm diameter silicon wafer at 500 r/min for 30 s using a spinner. It spread out as a thin sheet (0.1−0.2 mm thick) and then was cured overnight at 80°C. The sheet of cured PDMS was cut into small square (18 × 18 mm), and ultraviolet light was used for sterilization of the PDMS.

### Swarm Agar

Swarm agar (0.45% Eiken agar, 1% Bacto peptone, 0.3% beef extract, 0.5% NaCl) was prepared and then stored in sterile oven at 65°C. Tween 20 was added to a final concentration of 0.05% before use. Antibiotics were added at the concentrations used in liquid cultures, IPTG and arabinose were added to a final concentration of 2 mM and 0.5%, respectively if needed. Polystyrene Petri plates (150 mm diameter) were filled with 25 mL swarm agar and then cooled for 1 h (without a lid) inside a large Plexiglas box at 23°C. The plates were inoculated with a 2.5 μL drop of the overnight culture diluted by a factor of 10^3^ (for HCB1736/SM2/ZW1) or 10^5^ (for HCB1) with swarm medium (1% Bacto peptone, 0.3% beef extract, and 0.5% NaCl), placed at the center of the Petri plates. The plates were dried for another 30 min, covered, and incubated for ∼18 h at 30°C in an incubator (100% relative humidity). In order to determine the spreading rate of the swarm, we marked the leading edge of the Petri dish every hour after 10 h of incubation.

### Tracking Bacteria on the Swarm Plate

Cells of HCB1 or HCB1736 carrying pTrc99amCherry were mixed with the corresponding cells not carrying the plasmid and inoculated on the swarm plate at a ratio of 1:50 (for tracking the cells in monolayer area) or 1:1000 (for tracking multilayer area) so that we can track the trajectory of individual cells (Ariel et al., 2015; Chen et al., 2017). After ∼18 h the swarm plate was put on a Nikon Ni-E upright fluorescence microscope equipped with a temperature controlled sample stage (maintaining temperature at 30°C). The swarm plates were covered with a lid to prevent evaporation. The microscope was equipped with a filter-set for mCherry and a sCMOS camera (C11440, Hamamatsu). Cells were imaged with epifluorescence at a frame rate of 25 fps with a 40 × dry objective (Nikon CFI S Plan Fluor ELWD ADM 40 ×, NA 0.6, WD 2.8–3.6 mm).

### Labeling Flagella

Swarm cells were collected by gently rinsing the swarm monolayer region with 1 mL of motility medium (10 mM potassium phosphate, 0.1 mM EDTA, 10 mM lactic acid, and 70 mM NaCl, at pH 7.5) from four Petri plates. Then the cells (both swarm cells and cells grown in bulk liquids) were labeled following the protocol described previously (Turner et al., 2010). Cells were washed three times by centrifugation (2000 × *g*, 10 min) and gentle resuspension in 1 mL of motility medium. The final pellet was adjusted to a volume of ∼100 μL which concentrated 10-fold. For SM2 transformed with pBAD33FliC^S353C^, 4 μL solution of Alexa Fluor 568 maleimide [Invitrogen-Molecular Probes, 5 mg/mL in dimethyl sulfoxide (DMSO)] was added, and labeling was allowed to proceed for 60 min at room temperature, with gyro rotation at 100 r/min. Then unused dye was removed by washing cells with motility medium three times, and cells were suspended to a final volume of ∼200 μL for addition to the monolayer region on swarm plate or ∼2.0 mL for addition to the tunnel between the coverslip and a glass slide (see section “Fluorescence Imaging”).

### Fluorescence Imaging

Coverslips were coated with poly-L-lysine, and a tunnel was formed with two pieces of double-sided tape spaced between the coverslip and a glass slide. To count the number of flagella per cell, 40 μL of labeled cells were placed on the glass coverslip coated with poly-L-lysine and allowed to stand for 5 min and then the tunnel was rinsed with 100 μL motility medium. Before observation, cells were pre-processed with sodium azide solution (50 mM) in order to stop the flagellar rotation.

To visualize the dynamic behavior of flagella, cells were imaged in a sandwich-like setting between a glass coverslip and a thin sheet of PDMS described previously (Turner et al., 2010). Swarm cells from the monolayer region were labeled and added (∼2 μL drop) to the monolayer region in another swarm plate. The swarm plate was returned to the 30°C humid incubator for another 1 h. A square PDMS sheet (see section “PDMS Sheet”) was placed at the region near the drop point. The PDMS sheet was lifted, removing the bacteria by blotting as described previously (Darnton et al., 2004; Turner et al., 2010), and then was placed cell side down on a rectangular coverslip (24 × 60 mm). Another coverslip (24 × 32 mm) was put on top of the PDMS sheet to reduce evaporation. Each preparation was used for 20 min.

Both the tunnel-like and sandwich-like settings were put on a Nikon Ti-E inverted fluorescence microscope with the filter-set for fluorescein, a 100 × oil-immersion objective (Nikon CFI Apo Lambda DM 100 × Oil, NA 1.45, WD 0.13 mm), and a sCMOS camera (Primer95B, Photometrics). Single image was captured with a 50 ms exposure time. The videos for flagellar dynamic behavior were recorded at 80 fps, using a 10 ms exposure time.

### Preparation of Cell Suspensions in Quasi-Two-Dimensional Water Layer

Cells were collected from the day-culture solution, adjusted to the desired cell density. One end of the PDMS sheet was attached to a glass coverslip (24 × 60 mm) and then the appropriate amount of cell suspension was added to the glass coverslip. The PDMS sheet was flattened on the coverslip and another glass coverslip (24 × 32 mm) was placed over it to prevent evaporation. A thin layer of cell suspension was created between the PDMS sheet and the lower glass coverslip, and quasi-two-dimensional regions were often found near the edges.

### Data Analysis

Bacterial trajectories were analyzed with custom scripts in MATLAB. Trajectories were smoothed using MATLAB’s function “Sgolayfilt” which fits a polynomial (third order) to a moving window (11 frames). We extracted the positions of each bacterium after image processing, then velocity and propulsion angle (the acute angle between the body axis and the cell velocity vector) could be calculated. The “turning points” on the trajectories were defined as an instant with angular speed larger than a certain threshold (15, 30, and 45 rad/s were tried). The duration between two consecutive turning points is the waiting time. Trajectories longer than 10 s were used to analyze MSD and the waiting times. We confirmed the reversal event (cell head became tail) by eyes for individual trajectories. The reversal probabilities were calculated by dividing the number of reversals by the total duration of the trajectories (Turner et al., 2010). ImageJ software was used for measuring cell body length and flagellar length.

## Data Availability Statement

The datasets generated in the current study are available from the corresponding authors on reasonable request.

## Author Contributions

JY and RZ designed the work. ZW and RH performed the measurements. All authors wrote the manuscript.

## Conflict of Interest

The authors declare that the research was conducted in the absence of any commercial or financial relationships that could be construed as a potential conflict of interest.

## Supplementary Material

FIGURE S1 Photo images of a HCB1736 (Δ*cheY*) swarm colony at different Tween 20 concentrations. **(A–F)** Concentrations of 0, 0.01, 0.05, 0.1, 0.5, and 1%, respectively. Plates were photographed at 18 h after inoculation.

FIGURE S2 Photo images of a swarm colony with (right panels) or without (left panels) 0.05% Tween 20. **(A,B)** HCB1 (wildtype). **(C,D)** HCB1736 (Δ*cheY*). **(E,F)** ZW1 (Δ*motB*). Plates were photographed at 14 h after inoculation.

FIGURE S3 **(A)** A typical pattern of HCB1736 (Δ*cheY*) swarm on the agar plate with 0.05% Tween 20. **(B–D)** Snapshots of monolayer region, transition region, and multilayer region.

FIGURE S4 Cells in monolayer were longer on average, about twice the length of cells in multiple layers and cells grown in TB solution. **(A–C)** The distribution of cell length for the Δ*cheY* swarmer cells in the monolayer region, the multilayer region, and the Δ*cheY* swimmer cells grown in TB solution, respectively.

FIGURE S5 The average number and length of flagellar filaments for the Δ*cheY* swarmer cells in the monolayer region and those grown in TB solution were comparable. **(A,C)** Swarmer cells in the monolayer region. **(B,D)** The swimmer cells grown in TB solution.

FIGURE S6 Δ*cheY* cells in the monolayer region and the multilayer region and the wildtype cells in the monolayer region show the similar average velocity. **(A–C)** The distribution of cell velocities of the Δ*cheY* swarmer cells in the monolayer region, the multilayer region, and the wildtype swarmer cells in the monolayer region, respectively.

FIGURE S7 Δ*cheY* cells in the monolayer region and the multilayer region and the wildtype cells in the monolayer region show the similar average propulsion angle. **(A–C)** The distribution of the propulsion angles of the Δ*cheY* swarmer cells in the monolayer region, the multilayer region, and the wildtype swarmer cells in the monolayer region, respectively. The right panel shows the definition of the propulsion angle, with the red dot denoting the head of the cell.

FIGURE S8 Growth curves for *E. coli* strains HCB1 (wildtype) and HCB1736 (Δ*cheY*) in TB solution (200 r/min shaker, 30°C). Two strains showed near-identical growth rate.

FIGURE S9 Mean squared displacements of HCB1 (wildtype, blue circles) and HCB1736 (Δ*cheY*, red circles), both exhibiting normal diffusive behavior, with linear relationship between MSD and time.

FIGURE S10 Distribution of waiting times between turns showing an exponential shape regardless of the choice of cutoff angular speed. **(A,B)** The result of HCB1 (wildtype) and HCB1736 (Δ*cheY*), respectively. The solid lines represent the results of exponential fitting.

FIGURE S11 Distribution of the reversal angles for HCB1736 (Δ*cheY*) swarming.

MOVIE S1 A*cheY* mutant swarmer cell reversed its moving direction without changing the orientation of its body axis.

MOVIE S2 A*cheY* mutant swarmer cell exhibited successive reversals.

MOVIE S3 A *cheY* mutant swarmer cell exhibited a reversal accompanied by a significant rotation of the cell body.

MOVIE S4 Visualization of flagellar dynamics in a *cheY* mutant swarmer cell during a reversal with body rotation. Video was recorded at frame rate of 80 fps and played at 25 fps.

MOVIE S5 Visualization of flagellar dynamics in a *cheY* mutant swarmer cell during a reversal without much body rotation. Video was recorded at frame rate of 80 fps and played at 25 fps.

MOVIE S6 A typical reversal of a *cheY* mutant swimming cell in a quasi-two-dimensional water layer.

MOVIE S7 Visualization of flagellar dynamics in a *cheY* mutant swimming cell during a reversal in a quasi-two-dimensional water layer. Video was recorded at frame rate of 80 fps and played at 25 fps.
