# Supplementary figures and images for: Swarming Motility Without Flagellar Motor Switching by Reversal of Swimming Direction in E. coli
Source: Front Microbiol. 2020 May 21;11:1042. doi: 10.3389/fmicb.2020.01042 (PMC7326100; doi:10.3389/fmicb.2020.01042)

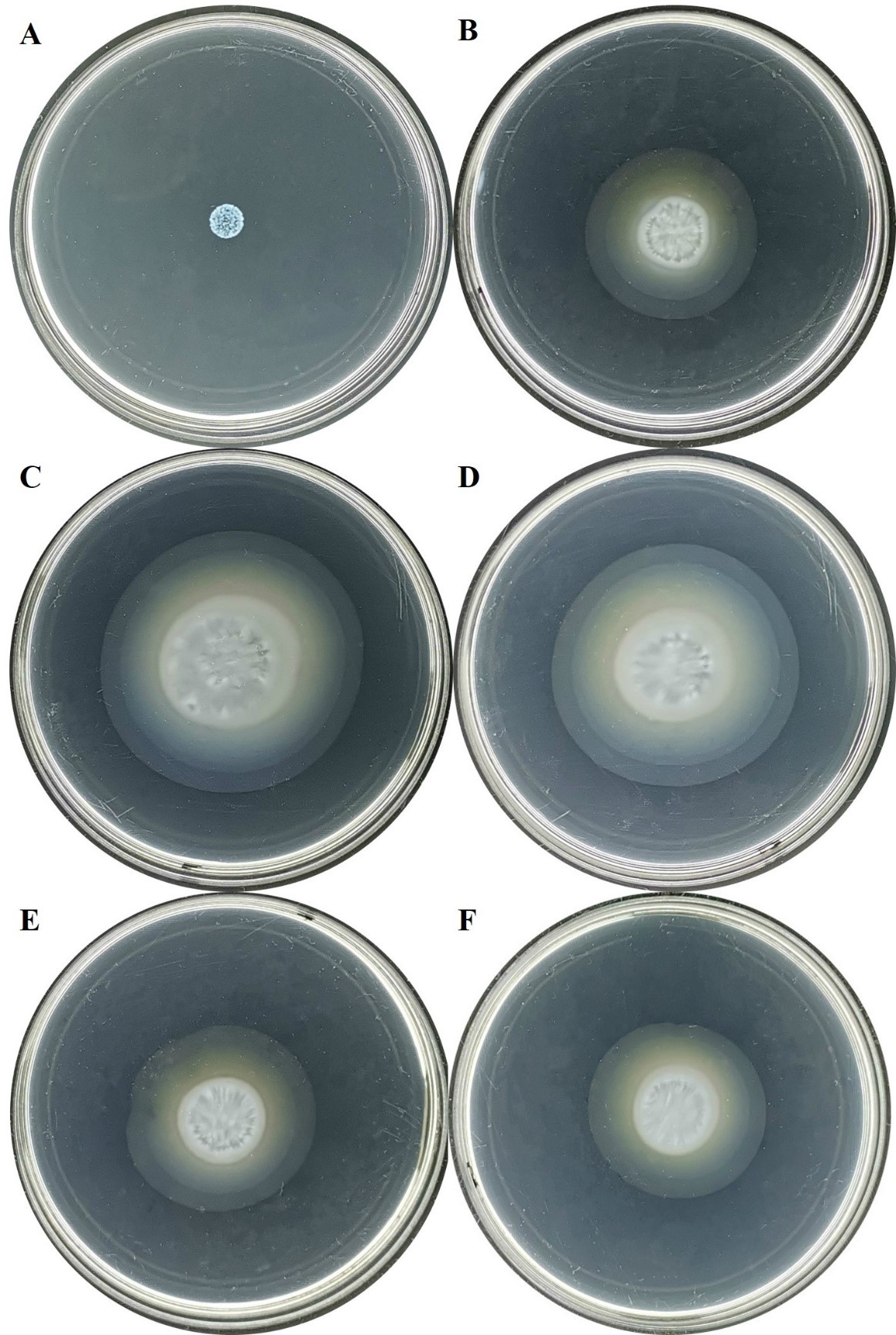

Supplement: FIGURE S1 — Photo images of a HCB1736 (ΔcheY) swarm colony at different Tween 20 concentrations. (A–F) Concentrations of 0, 0.01, 0.05, 0.1, 0.5, and 1%, respectively. Plates were photographed at 18 h after inoculation. [file Image_1.jpg]

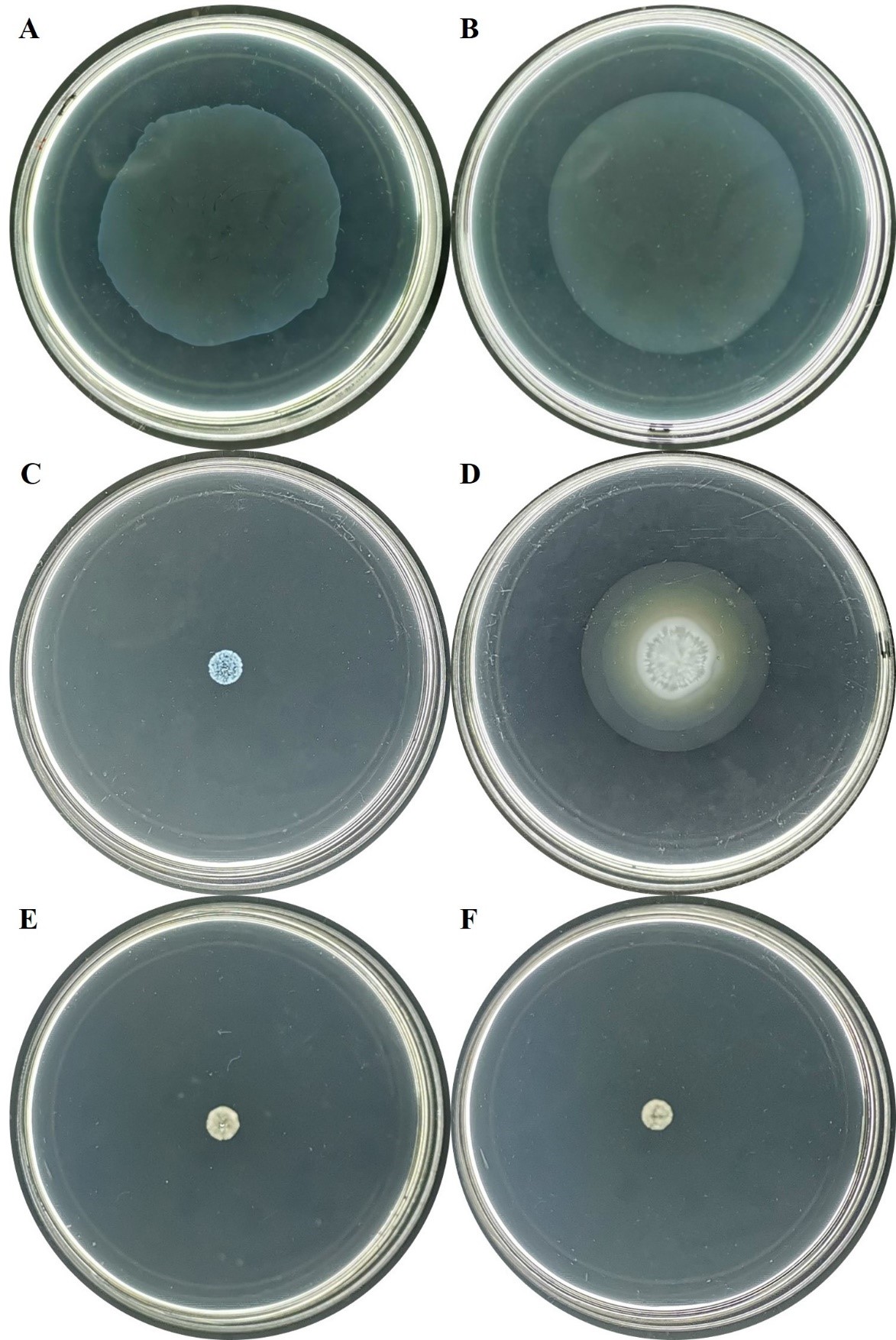

Supplement: FIGURE S2 — Photo images of a swarm colony with (right panels) or without (left panels) 0.05% Tween 20. (A,B) HCB1 (wildtype). (C,D) HCB1736 (ΔcheY). (E,F) ZW1 (ΔmotB). Plates were photographed at 14 h after inoculation. [file Image_2.jpg]

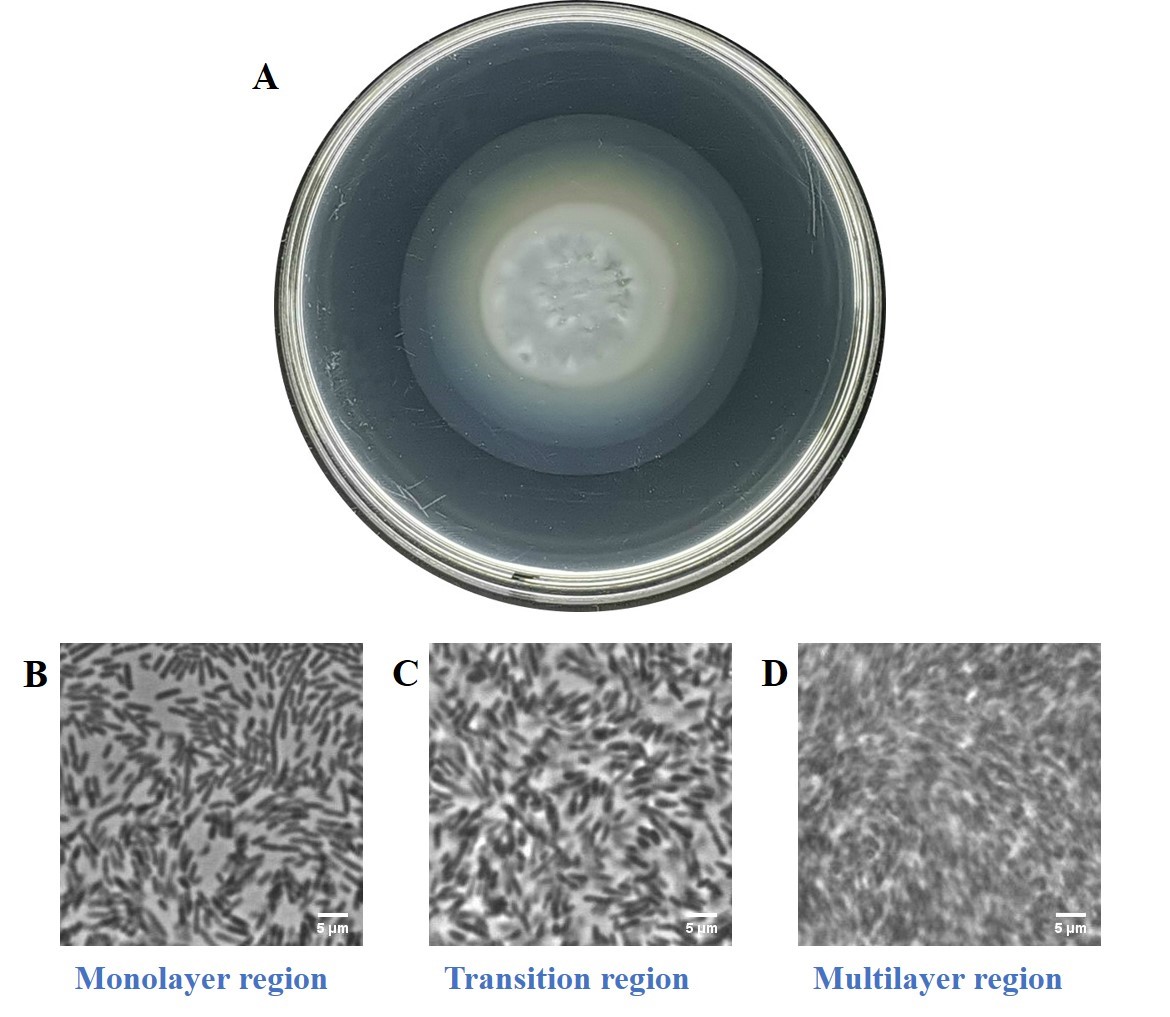

Supplement: FIGURE S3 — (A) A typical pattern of HCB1736 (ΔcheY) swarm on the agar plate with 0.05% Tween 20. (B–D) Snapshots of monolayer region, transition region, and multilayer region. [file Image_3.jpg]

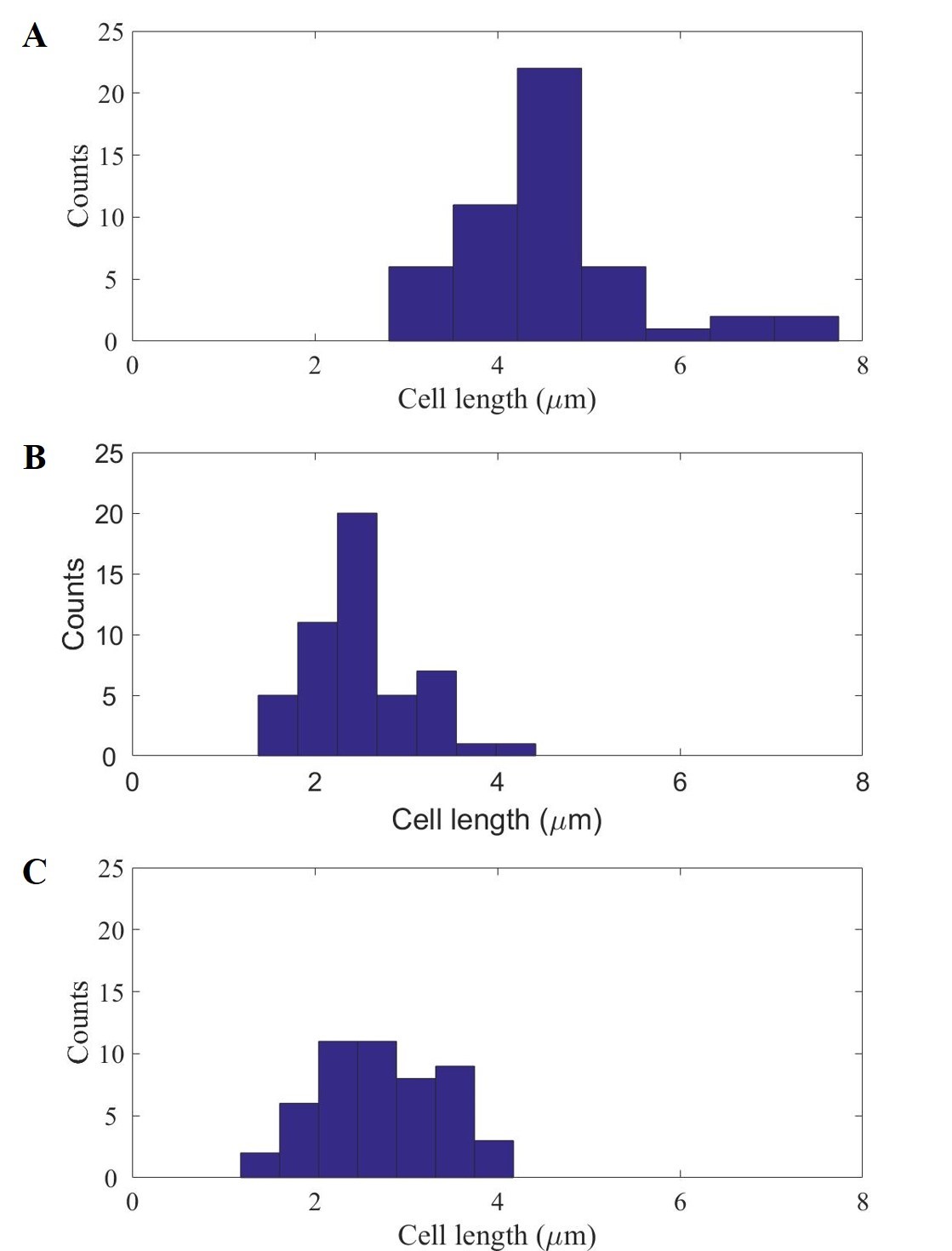

Supplement: FIGURE S4 — Cells in monolayer were longer on average, about twice the length of cells in multiple layers and cells grown in TB solution. (A–C) The distribution of cell length for the ΔcheY swarmer cells in the monolayer region, the multilayer region, and the ΔcheY swimmer cells grown in TB solution, respectively. [file Image_4.jpg]

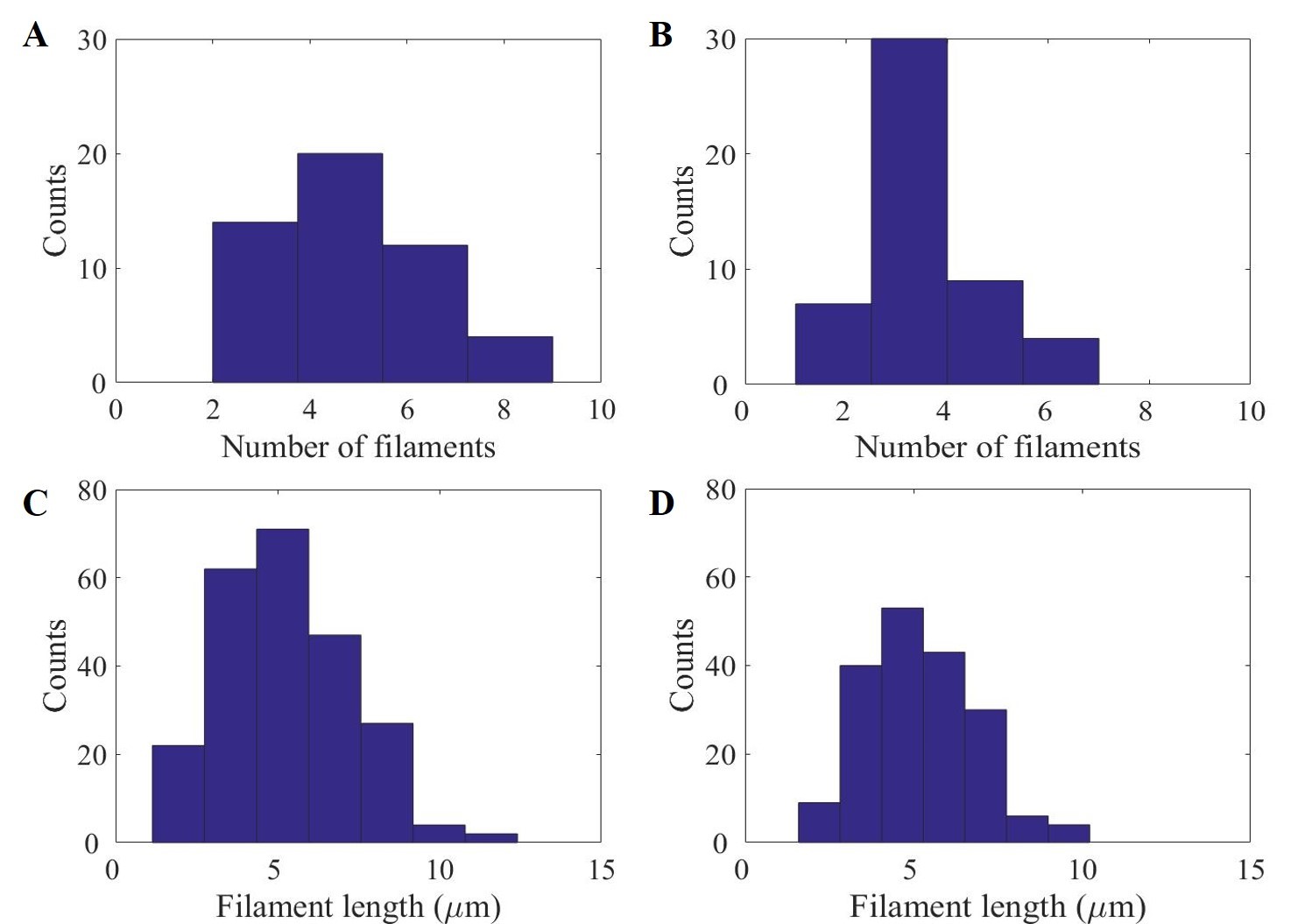

Supplement: FIGURE S5 — The average number and length of flagellar filaments for the ΔcheY swarmer cells in the monolayer region and those grown in TB solution were comparable. (A,C) Swarmer cells in the monolayer region. (B,D) The swimmer cells grown in TB solution. [file Image_5.jpg]

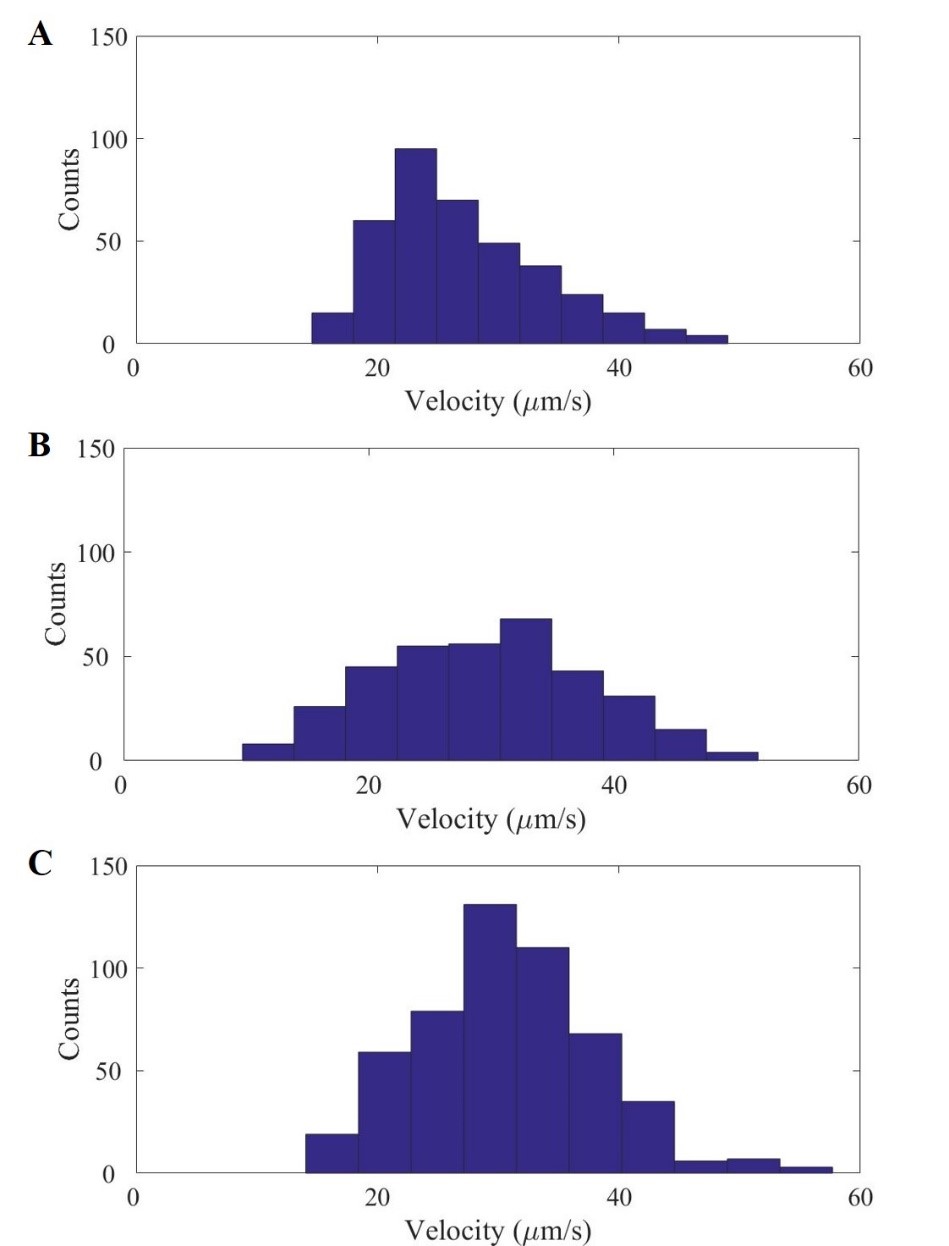

Supplement: FIGURE S6 — ΔcheY cells in the monolayer region and the multilayer region and the wildtype cells in the monolayer region show the similar average velocity. (A–C) The distribution of cell velocities of the ΔcheY swarmer cells in the monolayer region, the multilayer region, and the wildtype swarmer cells in the monolayer region, respectively. [file Image_6.jpg]

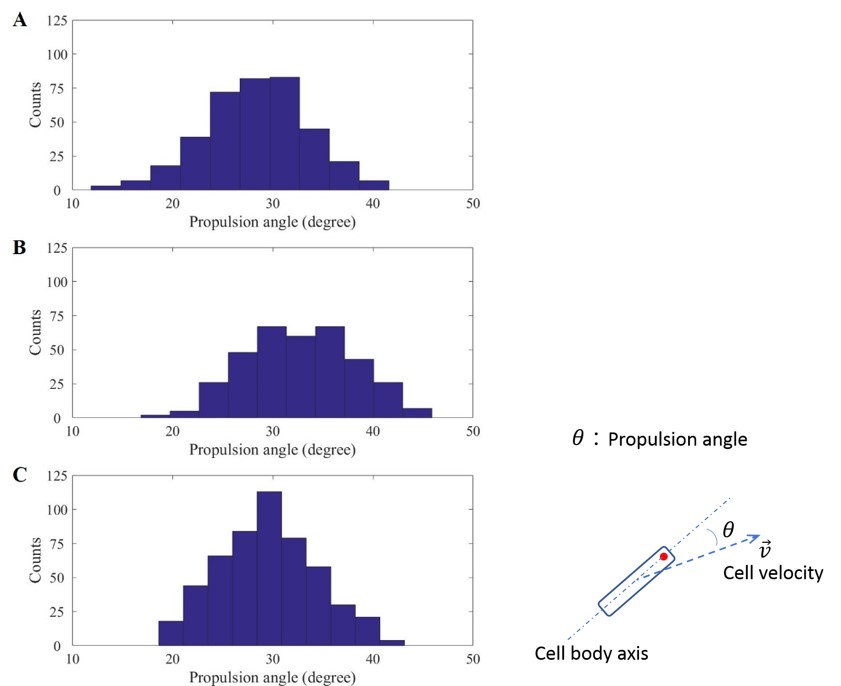

Supplement: FIGURE S7 — ΔcheY cells in the monolayer region and the multilayer region and the wildtype cells in the monolayer region show the similar average propulsion angle. (A–C) The distribution of the propulsion angles of the ΔcheY swarmer cells in the monolayer region, the multilayer region, and the wildtype swarmer cells in the monolayer region, respectively. The right panel shows the definition of the propulsion angle, with the red dot denoting the head of the cell. [file Image_7.jpg]

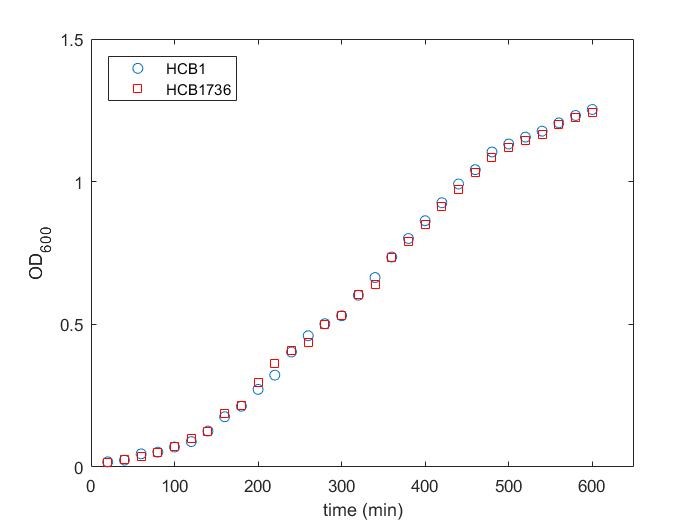

Supplement: FIGURE S8 — Growth curves for E. coli strains HCB1 (wildtype) and HCB1736 (ΔcheY) in TB solution (200 r/min shaker, 30°C). Two strains showed near-identical growth rate. [file Image_8.jpg]

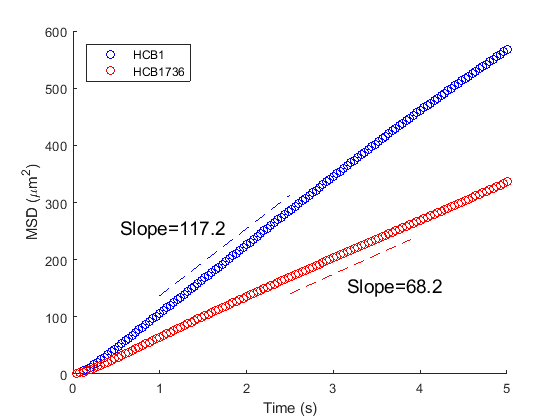

Supplement: FIGURE S9 — Mean squared displacements of HCB1 (wildtype, blue circles) and HCB1736 (ΔcheY, red circles), both exhibiting normal diffusive behavior, with linear relationship between MSD and time. [file Image_9.tif]

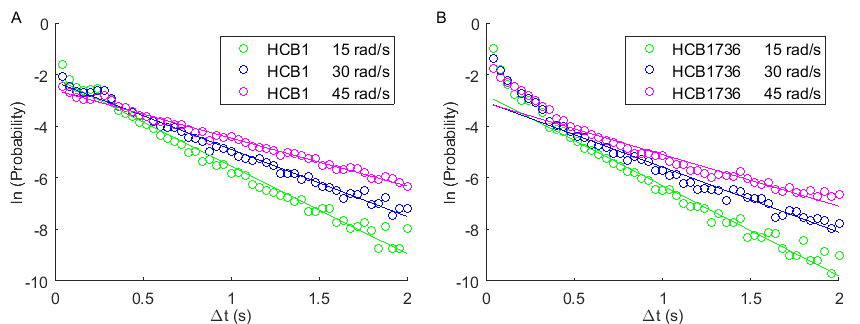

Supplement: FIGURE S10 — Distribution of waiting times between turns showing an exponential shape regardless of the choice of cutoff angular speed. (A,B) The result of HCB1 (wildtype) and HCB1736 (ΔcheY), respectively. The solid lines represent the results of exponential fitting. [file Image_10.tif]

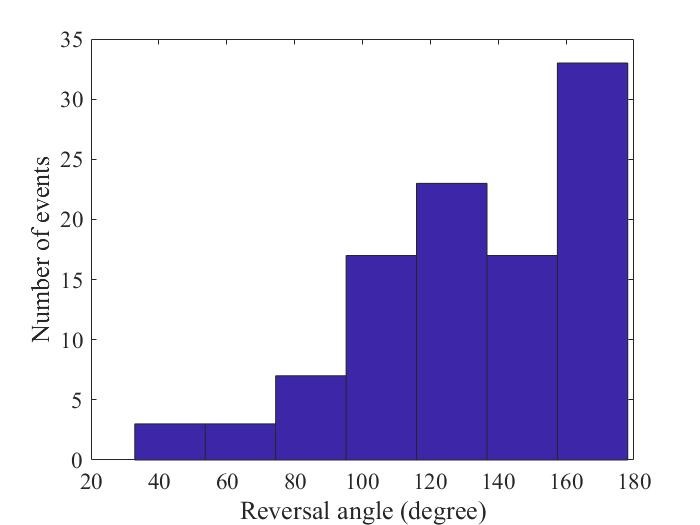

Supplement: FIGURE S11 — Distribution of the reversal angles for HCB1736 (ΔcheY) swarming. [file Image_11.jpg]
